# Radiological Manifestations of Rhino-Orbito-Cranial Mucormycosis in COVID-19 Patients Correlated With Pathological and Clinical Outcomes and Emphasis on Magnetic Resonance Imaging-Based Scoring System

**DOI:** 10.7759/cureus.35745

**Published:** 2023-03-03

**Authors:** Chandrasekhar Patil, Arun Kumar, Vasudha Battula, Prashanth Kumar, Raja Kollu, Sai Kotamraju, Bhavana Lakshmi Nethi Balingari, Sushmitha Reddy, Smitha Ravula, Akash R Reddy

**Affiliations:** 1 Radiodiagnosis, Malla Reddy Medical College for Women, Hyderabad, IND; 2 Radiodiagnosis, MaAx Super Speciality Hospital, Shimoga, IND; 3 Pediatric Intensive Care Unit, Ankura Hospital, Hyderabad, IND; 4 Radiology, Malla Reddy Medical College for Women, Hyderabad, IND; 5 Internal Medicine, Bhaskara Medical College, Hyderabad, IND

**Keywords:** saturated short tau inversion recovery sequence (stir), invasive fungal sinusitis, cerebral sinus venous thrombosis (csvt), black turbinate sign, mucormycosis

## Abstract

There was tremendous increase in the number of cases of mucormycosis among patients affected by coronavirus disease 2019 (COVID-19) during the second wave of pandemic in South Asian countries. This invasive fungal infection primarily affects paranasal sinuses and can have orbito-facial and intracranial extension. We are presenting the radiological findings of invasive mucormycosis with pathological and clinical outcome correlation. It is important for radiologists to have the knowledge of various presentations of this opportunistic infection for early diagnosis and helping clinicians in planning the appropriate line of management. The study also emphasizes on the correlation between the extent of involvement with clinical outcome and we proposed a magnetic resonance imaging (MRI) based scoring system to standardize and prognosticate the patients affected with mucormycosis.

Materials and methods: We utilized GE 1.5 tesla, 16-channeled MRI machine for scanning the clinically suspected mucormycosis patients and did plain and contrast study of the paranasal sinuses, orbito-facial study and included brain as and when required. Images were acquired in axial, coronal, and sagittal planes using T1, T2, and fat-saturated short tau inversion recovery sequences (STIR), fat-saturated contrast sequences for better evaluation of the extent of the disease. Diffusion-weighted sequence was also acquired to detect ischemic changes in optic nerve or brain parenchyma. Contrast study was used to detect any major vessel occlusion or cavernous sinus thrombosis in the study population.

Results: Total number of cases (n) included in the study were 32. The mean age group was 41-50 years with the median age was 47 years. Out of 32 cases (n=32), in 16 cases (50%) the disease was limited only to the paranasal sinuses and in remaining 16 (50%) cases, disease has spread to other regions such as orbits, facial soft tissues, optic nerve, and brain parenchyma. All the 18 cases with Mild score (MRI ROCM score 1-3) survived and all those with severe score (2 cases) (MRI ROCM score 7-10) did not survive.

Conclusion: During the second wave of COVID-19 pandemic, we observed a signiﬁcant rise in acute invasive mucormycosis infection primarily involving the paranasal sinuses and spread to orbito-facial, cerebral parenchyma causing related complications and hence increased morbidity and death. Radiologically, using MRI, it was effectively possible to detect early extrasinonasal spread and other fatal complications thereby guiding the physicians and surgeons in the proper early aggressive management of the disease. Here, we have described the radiological characteristics of paranasal sinus mucormycosis and its spread to other regions. We also proposed an MRI-based Scoring System for standardized assessment of the disease severity. We observed in our study that the extent of disease on MRI is directly correlating with mortality.

## Introduction

Mucormycosis (previously known as zygomycosis) is a serious but rare fungal infection caused by a bunch of molds known as mucormycetes. These molds live throughout the surroundings. This angioinvasive fungal infection commonly affects individuals with low immunity, elderly especially chronic diabetics, and individuals with chronic steroid or iatrogenic immunosuppression and patients with hematological malignancies, and those who have undergone organ transplantation use [1]. It most typically affects the sinuses or the pulmonary tissue through inhalation of fungal spores. Examples of fungi causing mucormycosis are Rhizopus species, Mucor species, Rhizomucor species, Syncephalastrum species, Cunninghamella bertholletiae, Apophysomyces species, and Lichtheimia (formerly Absidia) species [2].

During the second wave of the coronavirus disease 2019 (COVID-19) pandemic in India, there was an increased incidence of mucormycosis infection in COVID-19 patients due to various factors. Rampant steroid use, broad-spectrum antibiotics use, diabetes, use of unsterile oxygen masks/ventilators, and lack of awareness/hygiene could have contributed to the increased incidence.

This invasive fungal infection most often primarily affects paranasal sinuses then spreads to orbits, brain, and facial skeleton and causes orbital cellulitis, optic neuritis, encephalitis, or infarction. It is more aggressive with rapid progression and may also show recurrence in cases of incomplete surgical debridement or improper medical management. It may also cause death due to complications such as intracranial hemorrhage. Mortality rates due to intraorbital and intracranial complications of mucormycosis range from 50% to 80% [3]. Hence, early diagnosis is crucial for appropriate management in treating patients, reducing complications, and for good clinical outcomes.

The clinical symptoms depend on the site of involvement and common presenting symptoms which include nasal blockade, proptosis, facial pain and edema, ptosis, chemosis, anesthesia/decreased sensation over affected face, and even ophthalmoplegia. In cases of intracranial extension, headache, altered sensorium, fever, and various other neurological signs and symptoms can be noted [4,5].

The study is conducted in the Department of Radiology, Malla Reddy Narayana Multispeciality Hospital, Suraram, Hyderabad. Here we present magnetic resonance imaging (MRI) findings of paranasal sinuses, orbital and cerebral mucormycosis. Follow-up of these cases for clinical outcome and correlation with biopsy was done to the extent possible.

The methods available to detect or diagnose mucormycosis infection are multidetector computed tomography (MDCT), MRI, laboratory analysis such as biopsy, and microscopic analysis. MDCT and MRI are non-invasive and key imaging modalities available not only for diagnosis but also for detecting disease extension and its complications thereby significantly helping in guiding proper management.

## Materials and methods

We used GE 1.5 tesla, 16-channeled magnetic resonance imaging (MRI) machine (Chicago, IL: GE HealthCare) for scanning patients, referred for suspected mucormycosis, and did both plain and contrast study of paranasal sinuses, orbito-facial study, and included brain as and when required. Images were acquired in axial, coronal, and sagittal planes using T1, T2, and fat-saturated short tau inversion recovery sequences (STIR), and fat-saturated contrast sequences for better evaluation of site and extent of disease. Diffusion-weighted sequence was also acquired to detect ischemic changes in optic nerve or brain parenchyma. Contrast study helped to detect cavernous sinus thrombosis in our study.

Inclusion and exclusion criteria

We included both COVID-19 and post-COVID-19 patients having clinical suspicion of mucormycosis fungal infection and referred for paranasal sinus, brain, and orbit imaging in the period between April 2021 and June 2021. Cases outside the study period were not included, including non-COVID-19 patients.

## Results

Our study includes a total of 32 cases (n=32) out of which the clinical outcome of 23 cases was followed up while the clinical outcome of nine cases could not be followed up. Most common age group in our study was 41-50 years and the 21-30 years age group had the least number of cases (Table 1). Out of 32 cases, nine cases were females and remaining 23 cases were males (Table 1). In our study, 24 cases were diabetic, four cases had chronic kidney disease, and three cases did not have any co-morbidities (Table 1).

**Table 1:** Demographic data and comorbidities.

Variables		Number of individuals	Percentage
Age (years)	21-30	1	3.1
	31-40	9	28.1
	41-50	12	37.5
	51-60	6	18.8
	61-70	0	0.0
	71-80	4	12.5
Total		32	-
Sex	Males	23	71.9
	Females	9	28.1
Comorbidities	Diabetes mellitus	24	75
	Chronic kidney disease	4	12.5
	No comorbidities	4	12.5

Major pattern of sinus involvement was mucosal thickening while other patterns were partial or complete opacification of sinuses, air-fluid levels, and air bubbles within fluid levels. In our study, isolated sinonasal involvement was eight cases while 24 cases had multiple sinus involvement (Table 2). Contrast studies had different types of enhancement patterns, such as homogenous, heterogeneous, and few cases didn’t show any enhancement at all which is highly suggestive of necrosis. In our study, there were two cases of black turbinate sign and one case of maxillary sinus fungal ball.

**Table 2:** Sinonasal and extrasinonasal involvement.

Variables		Number of cases	Percentage
Sinonasal involvement	Isolated single sinus	8	25
	Multiple/pan sinusitis	24	75
Extrasinonasal involvement (27 cases)	Orbital, periorbital, and maxillofacial soft tissue extension	11	40.7
	Brain	4	14.8
	Bone	9	33.3
	Optic nerve	3	11.1

Extrasinonasal involvement was noted in 27 cases, of which 11 cases had orbital, periorbital, and maxillofacial soft tissue involvement; in three cases, the optic nerve was involved in the form of thickening with diffuse signal changes and/or diffusion restriction of optic nerve with perioptic soft tissue inflammation, brain is involved in four cases. There were five cases of marrow edema involving different bones like alveolar process of maxilla, ramus of mandible, and sinus wall, and a few cases with bony destruction/erosion of sinus walls and orbital walls (Table 2).

In our study with the available data, we proposed an MR imaging-based scoring system to unify and standardize the reporting findings and grading and categorizing accordingly, but needs more studies and future research to validate.

Defining the MRI-based scoring system

Since its high contrast resolution and advance in sequences, especially T2 fat-saturated (FAT-SAT)/STIR sequence, MRI is superior to CT in detecting subtle extrasinonasal soft tissue extension like early infiltration into brain or orbit, and also optic nerve involvement. Marrow edema is also better appreciated in MRI much before evident on CT as bone lysis/destruction.

In this scoring system we included paranasal sinus involvement, extrasinonasal spread, such as to the orbit, maxillofacial soft tissue, optic nerve, brain, and bone involvement assigned the score as depicted in Table 3, and graded into mild, moderate, and severe as depicted in Table 4.

**Table 3:** Proposed MRI-based ROCM scoring system. MRI: magnetic resonance imaging; ROCM: rhino-orbito-cranial mucormycosis

Sinonasal and extrasinonasal involvement	Score
Sinus involvement	+1
Bone marrow edema	+2
Orbital, periorbital, and maxillofacial soft tissue extension	+1
Optic nerve	+2 (+1 for each optic nerve)
Intracranial extension	+4
Total MRI ROCM score	10

**Table 4:** MRI-based ROCM grading system. ROCM: rhino-orbito-cranial mucormycosis

Score	Grade
1-3	Mild
4-6	Moderate
7-10	Severe

Highest individual score has been assigned to intracranial extension as it majorly influences the severity and clinical outcome. Our scoring system has a maximum score of "10" which is simple, fast tracking, and easier to implement in emergency situations like the recent COVID-19-associated mucormycosis surge. Based on our proposed MRI-based scoring system, 24 (75%) cases were mild, six (18.8%) cases were moderate, and two (6.3%) cases were severe in our study population (n=32) (Table 5).

**Table 5:** Proposed ROCM grading system implemented in our study population (n=32). ROCM: rhino-orbito-cranial mucormycosis

Score grade	Number of cases	Percentage
Mild	24	75.0
Moderate	6	18.8
Severe	2	6.3

We were able to follow-up 23 out of 32 cases and observed that 18 cases had clinically improved while five cases succumbed to the disease. Of the five deaths observed, three cases were grouped as moderate and two cases as severe based on the above-proposed MRI-based scoring system. The remaining followed up 18 cases had clinically improved and were grouped as mild form. In our study, the mild form (score 1-3) showed significant clinical improvement and the severe form (score 7-10) succumbed to the disease (Table 6).

**Table 6:** ROCM grade in the study population correlated with the clinical outcome. ROCM: rhino-orbito-cranial mucormycosis

ROCM score grade	Number of individuals	Clinically improved	Death
Mild	24	24	0
Moderate	6	3	3
Severe	2	0	2

## Discussion

Mucormycosis spreads through the nasal route, often the nasal or paranasal sinuses are the primary sites of involvement thereby spreading to the para-antral soft tissues, facial bones, base of skull, and central nervous system. In addition to diffuse alveolar injury with severe inflammatory exudation, COVID-19 patients have immunosuppression due to decrease in CD4+ and CD8+ T cells [6].

Multiple reasons were postulated for the increase in incidence of mucormycosis in patients with COVID-19 during the second wave of the COVID-19 pandemic in India. The reasons mentioned in the previous literature are as follows: (1) immunosuppression caused by the COVID-19 infection itself which poses additional risk in patients with other comorbidities, (2) hyperferritinemic syndrome which is seen in severe COVID-19 disease that poses a risk for cellular damage and increased free iron that ultimately increases risk for acquiring mucormycosis [7,8], (3) hyperglycemia due to uncontrolled pre-existing diabetes, (4) high prevalence rates of mucormycosis in India per se, (5) rampant overuse and irrational use of steroids in the management of COVID-19, (6) prolonged ICU stay, and (7) irrational use of broad-spectrum antibiotics. Pre-existing comorbidities, such as solid organ transplant, hematological malignancies, use of immunosuppressants, etc., also were the other mentioned risk factors.

Spread to orbit is thought to be via the nasolacrimal duct and erosion of medial orbital wall, congenital dehiscence in medial wall of orbit, and also through the arterial or venous perforators in the medial wall of orbit. Such invasion is facilitated by the thinness of the lamina papyracea, congenital dehiscence often present along the medial wall, and the perforations of the medial wall by arteries and veins [9,10].

Mucormycosis is an angioinvasive fungus, its spread to brain is thought to occur from angioinvasion causing occlusion/thrombosis of vessels leading to cerebral infarction and dissemination to central nervous system [11-13]. Spread through cribriform plate and orbital apex are also possible mechanisms of central nervous system involvement [14].

The presenting symptoms vary depending on the site of involvement but most often fever, pain and facial swelling, blurring of vision, cranial nerve palsies from cavernous sinus involvement, and even loss of vision or paraparesis from cerebral infarction would be the presenting symptoms. However, it is a fatal fungal disease with rhino-cerebral manifestations being the most common [10].

Cavernous sinus thrombosis is well known vascular complication of this fungus and occurs from the orbit, across the orbital apex, and on MRI the cavernous sinus thrombosis appears as loss of flow void on T1- and T2-weighted imaging and absent enhancement on contrast images [15].

Mycotic aneurysms are another form of vascular complications of mucormycosis in addition to thrombo-occlusion [16]. Vascular invasion causing thrombosis of the cavernous segment of the internal carotid artery is the common event causing cerebral infarcts. The invasion of vessel is seen as arterial wall enhancement in post-contrast images [17]. Facial, orbital fat stranding, and retro-antral soft tissue extension are the early indicators of the aggressive course of the disease [18,19]. Extrasinonasal disease spread to pre-antral, facial, orbital, and cranial involvement are all features of acute invasive fungal sinusitis.

All patients suspected of rhino-cerebro-ocular mucormycosis received antifungal medication and underwent magnetic resonance imaging of paranasal sinuses (MRI-PNS) examinations. Few patients were primarily referred for computed tomography of paranasal sinuses (CT-PNS) followed by an MRI-PNS. Based on the MRI findings, endoscopy and biopsy were suggested.

All the suspected patients received different treatments and surgical procedures depending on the involvement and extension of the pathology. Few patients underwent endoscopic debridement of devitalized fungal infected tissue, few underwent inferior maxillectomy, while few underwent facial resection. Few patients with partial visual loss underwent orbital debridement.

Diagnostic tests available for confirmation are KOH (10-20% potassium hydroxide) staining, microscopy, histopathology of debrided tissue and culture, Grocott’s methenamine silver stain, and periodic acid Schiff (PAS) staining.

The study was conducted during the second wave of the COVID-19 pandemic, where there was increased prevalence of concomitant mucormycosis cases. So, the study emphasized on mucormycosis infection. However, few bacterial infections like Klebsiella can also cause similar aggressive necrotizing bacterial rhinitis with different pathological and microbiological profiles and these bacterial infections are usually sporadic in nature [20].

**Figure 1:**
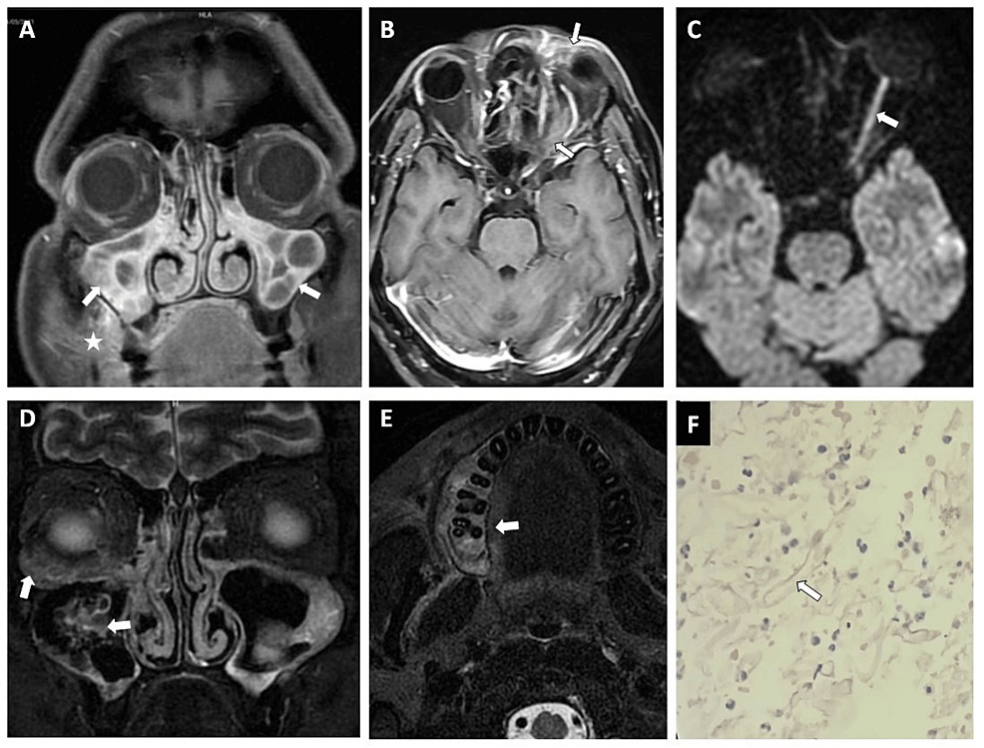
Case of mucormycosis in a post-COVID-19 patient having sinonasal and extrasinonasal spread. The patient underwent debridement and histopathological evaluation of the tissues was done. (A) Coronal T2WI showing mucosal thickening in bilateral maxillary sinus with lace-like heterogeneous enhancement (arrows) and also note the right peri-antral soft tissue enhancement (star) - s/o extrasinus extension. (B) Axial post-contrast image showing left orbital soft tissue inflammation (arrow) and periorbital soft tissue enhancement (arrow). (C) Same patient diffusion-weighted image (DWI) showing diffusion restriction in left optic nerve suggestive of optic nerve involvement. (D) Different case coronal short tau inversion recovery (STIR) image showing bilateral maxillary sinus involvement with frothy material in the right maxillary sinus (arrow). Note also right orbital soft tissue edema/ inflammation (arrow) - suggestive of orbital soft tissue and bone marrow spread of mucormycosis. (E) Same patient (as D) axial STIR image showing marrow edema in the alveolar process of the maxillary bone on the right side (arrow). (F) Hematoxylin and eosin (H&E) stained histopathological slide of the same patient showing non-septate widely branching hyphal elements. COVID-19: coronavirus disease 2019

**Figure 2:**
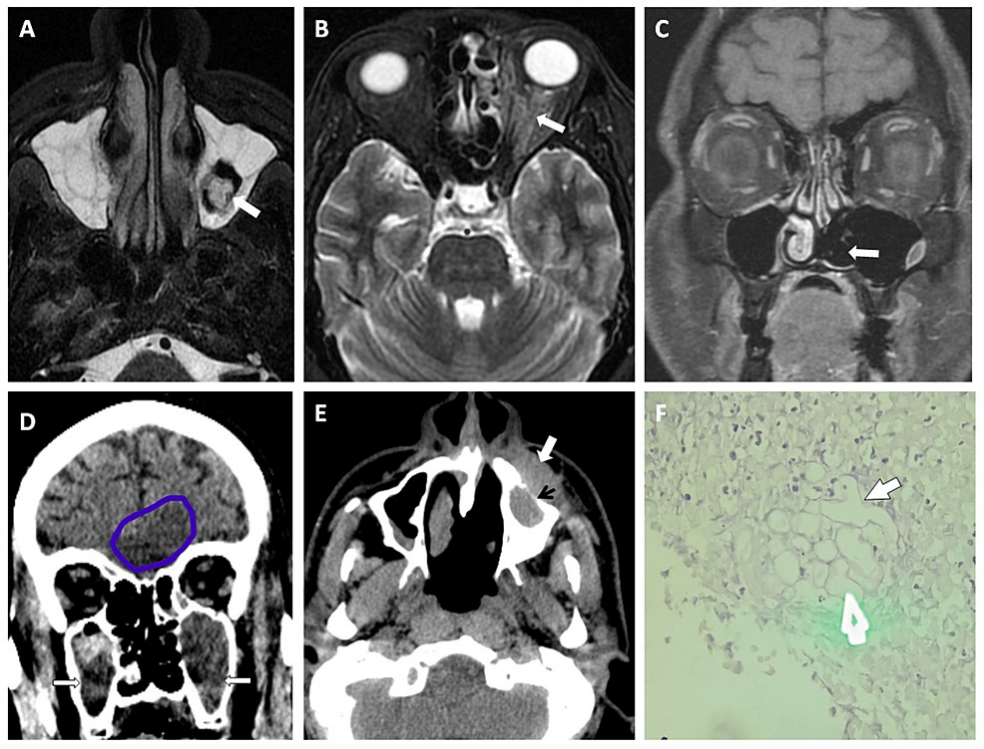
Two cases of post-COVID-19 diabetic patients having facial pain and decreased vision. (A) Axial T2 STIR image showing opacification of bilateral maxillary sinus with mildly hyperintense fungal ball in left maxillary sinus (arrow). (B) Axial T2 STIR image showing thickening with increased signal intensity seen in the left optic nerve with mild perioptic soft tissue edema (arrow), suggestive of optic neuritis. (C) Coronal fat-saturated (FAT-SAT) contrast image demonstrating non-enhancing left inferior turbinate whereas the right inferior turbinate shows normal enhancement suggestive of necrotic tissue (left black turbinate sign). (D) Coronal computed tomography (CT) image showing opacification of bilateral maxillary sinus (arrows) and a mild focal area of hypodensity in left basifrontal lobe (marked with blue circle) - suggestive of intracranial spread/encephalitis. (E) Axial CT image showing erosion of anterior wall of left maxillary sinus (black arrow) with pre-antral soft tissue thickening (white arrow). (F) Periodic acid Schiff (PAS) stained histopathological slide of the same patient showing non-septate widely branching hyphal elements infiltrating the tissues. COVID-19: coronavirus disease 2019

**Figure 3:**
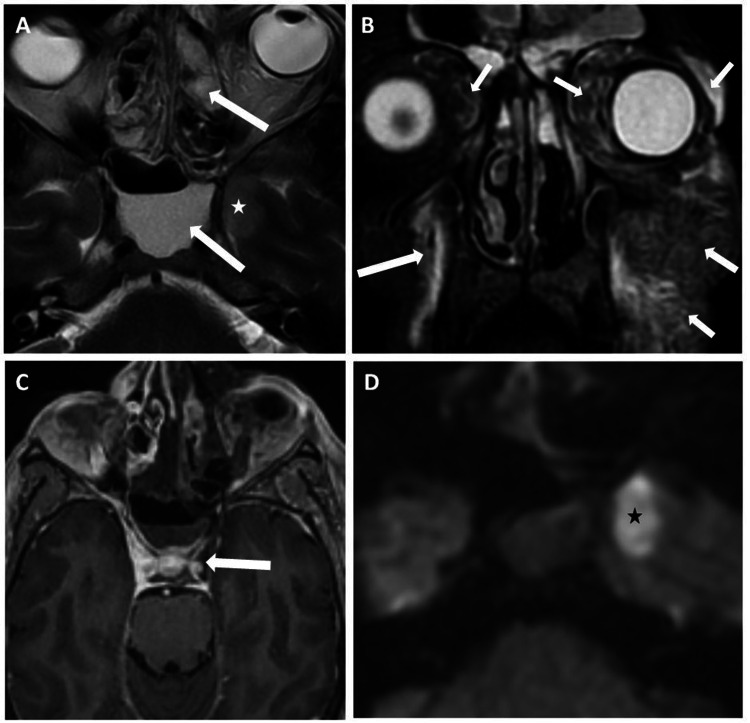
A case of complicated paranasal mucormycosis with extrasinonasal spread to bilateral orbits, para-antral maxillofacial soft tissue, and also left cavernous sinus thrombosis resulting in infarct in the left medial temporal lobe. (A) Axial T2 image showing severe mucosal thickening in bilateral ethmoid sinuses with air-fluid level in the sphenoid sinus (arrows). (Note: A focal hypodensity in the left medial temporal lobe (marked with white star). (B) Altered signal intensity/ edema noted in bilateral extrasinonasal orbital soft tissue and maxillofacial soft tissue (arrows) - suggestive of spread to orbital and maxillofacial soft tissue. (C) Axial T1 post-contrast image showing filling defect in the left cavernous sinus (arrow) causing infarct in the left temporal lobe (white star in A, black star in D). (D) Axial DWI image showing infarct in left temporal lobe (black star). COVID-19: coronavirus disease 2019

The following images demonstrate different cases of rhino-orbito mucormycosis with varied patterns of sinonasal involvement and extrasinonasal spread such as optic nerve involvement (Figures 1A-1F), intracranial spread (Figures 2A-2F), and cavernous sinus thrombosis (Figures 3A-3D).

White et al. conducted a study including 135 adults with COVID-19 infection and observed an incidence of 26.7% invasive fungal infections [21]. Song et al. studied the association between COVID-19 and invasive fungal sinusitis in April 2020 and showed that patients with COVID-19 infection or those who recovered from COVID-19 infection are at an increased risk of acquiring invasive fungal diseases [22].

Metwally et al. in their study published in 2022 used a diagnostic-reliable staging method to define the imaging features of post-COVID-19 head and neck mucormycosis and identify risk variables for extrasinus extension. They proposed an MR imaging-based classification of mucormycosis staging depending on the site and extent of involvement into stage 1 to stage 4, stage 1 is limited to sinonasal, stage 2 is extrasinus maxillofacial, stage 3 is intraconal and stage 4 is intracranial involvement. The prevalence of mucormycosis in stages 1, 2, 3, and 4 in our study was 11.1%, 55.6%, 20.6%, and 12.7%, respectively [23].

MRI imaging characteristics of rhino-orbtio-cranial mucormycosis

Features of sinusitis include mucosal thickening/opacification/fluid levels, frothy secretions, pan sinus involvement, and on MRI mucosal thickening appear T1-hypo and T2-hyper or -hypointense; fluid secretions appear as T2/STIR hyperintense. Intrasinus fungal elements may appear as low signal intensity on T2 sequences. Intrasinus fungal ball/mycetoma appears as irregular, spherical/mass-like intrasinus variable T1/T2 signal with convex margin often nonenhancing. Extrasinonasal soft tissue involvement appears hyperintense on FAT-SAT/STIR images and shows mild enhancement on contrast images.

Bone Involvement

Affected bone marrow appears hypointense on T1W and hyperintense on STIR images with heterogeneous post-enhancement [24].

Intracranial Extension

Involved areas appear T1-hypointense, T2/STIR hyperintense, and many times show diffusion restriction in cases of cerebral infarction by vessel occlusion and appear T1-hypointense, T2/FLAIR hyperintense, and may or may not show diffusion restriction.

Intraorbital Extension

Especially on STIR, hyperintensity in intraconal soft tissue, T2/STIR hyperintensity in pre-septal/post-septal orbital cellulitis, altered signal in optic nerve/perioptic region, optic nerve appear thickened, show diffusion restriction, and enhancement in post-contrast images in case of optic nerve involvement.

Contrast Enhancement

Three patterns are recognized in sinus involvement which are homogenous, patchy heterogeneous, and non-enhancing tissue. Non-enhancing tissue represents necrosed tissue, filling defect in cavernous sinus in cases of cavernous sinus invasion or thrombosis and occlusion of major vessels in cases of cerebral infarction.

Black turbinate sign, though not specific to mucormycosis, is also often found in rhino-nasal mucormycosis. Non-enhancement of turbinate (commonly middle or inferior) on contrast image suggesting necrosed tissue referred to as black turbinate sign which appears on endoscopy as black eschar, a sentinel sign of rhino-orbito-cerebral mucormycosis [25,26]. This back necrosed tissue is a result of local angioinvasion by the fungus with resultant thrombus and hence tissue infarction.

Limitations

Since the study included a smaller population, further large population studies are invited to collect more data in the future so as to standardize the reporting system and to further validate the proposed MRI-based ROCM scoring system.

## Conclusions

In our study, we observed the rampant raise of mucormycosis cases primarily involving the paranasal sinuses then contiguously spreading to the orbit, face, and brain causing devastating complications likely optic neuritis, encephalitis, brain infarctions, and even death.

With the use of available imaging information, we described the different types of MRI imaging characteristics of rhino-orbito-cranial mucormycosis and postulated the MRI-based scoring system to unify and standardize the reporting system and the study needs more data in the future for recommendation and validation.
